# Simulation of control scenarios of porcine reproductive and respiratory syndrome in Nghe An Province in Vietnam

**DOI:** 10.1111/tbed.13278

**Published:** 2019-07-07

**Authors:** Hu Suk Lee, Krishna K. Thakur, Vuong Nghia Bui, Anh Ngoc Bui, Minh Van Dang, Barbara Wieland

**Affiliations:** ^1^ International Livestock Research Institute (ILRI) Hanoi Vietnam; ^2^ Department of Health Management, Atlantic Veterinary College University of Prince Edward Island Charlottetown Canada; ^3^ National Institute of Veterinary Research Hanoi Vietnam; ^4^ Sub‐Department of Animal Health Tp Vinh Vietnam; ^5^ International Livestock Research Institute (ILRI) Addis Ababa Ethiopia

## Abstract

The main objective of this study was to develop various models using North American Animal Disease Spread Model (NAADSM) to simulate the transmission of Porcine reproductive and respiratory syndrome (PRRS) virus between farms in Nghe An Province in Vietnam in order to inform the prevention and control of this important disease. Using real data from the household survey, credible parameters for direct/indirect mean contact rates between different farms were estimated. A total of eleven models were developed, including immunization scenarios. In addition, we conducted sensitive analysis on how the mean contact rates influenced the results. The immunization scenarios showed that a high proportion of pigs in medium size farms needs to be vaccinated in order to reduce the transmission to pigs in small farms under the Vietnamese pig production system. In order to promote the use of vaccinations, incentives (such as a vaccine subsidy) for medium size farms may be needed. It could be the most cost‐effective control and prevention strategy for pig diseases in Vietnam. Our study provides insights on how pig diseases can be spread between pig farms via direct and indirect contact in Nghe An under the various hypothetical scenarios. Our results suggest that medium/large farms may play an important role in the transmission of pig diseases.

## 1 INTRODUCTION

Simulation models for evaluating the spread of contagious animal diseases are an important decision supporting tool for disease control (Keeling, 2005; Keeling et al., 2001; Morris, Sanson, Stern, Stevenson, & Wilesmith, 2002; Woolhouse, 2003), as demonstrated for example, during the Foot and mouth disease (FMD) outbreak in 2001 in the UK (Keeling et al., 2001; Taylor, 2003). Various modelling platforms have been developed, such as the North American Animal Disease Spread Model (NAADSM) a stochastic, spatial, farm‐level state‐transition modelling framework that was developed to simulate the spread of highly contagious diseases in animals (Harvey et al., 2007). The user‐established parameters define the disease transmission between farms, determined mainly by rates of direct contact, indirect contact and distances between farms.

In Vietnam, pork accounts for about 70% of all livestock products (Lich, 2001), with majority of pork produced by small‐scale farmers (GSO, 2016). Medium size pig farms (accounting for 20%–25%) are the main suppliers for piglets and weaners to small farms. The linkages between these different production systems and the large number of actors involved in pork production have implications for disease transmission. Intense contact between different farm movements of live animals between farms are considered the main route of disease transmission, which may lead to continuous emergence of epidemics (Gilbert et al., 2005).

To better understand the influence of contact patterns between farms on disease transmission and how this affects disease control, a simulation model was developed using porcine reproductive and respiratory syndrome (PRRS) as the disease example. In Vietnam, PRRS is endemic and has a major impact on production. It is a viral disease in pigs caused by a single‐stranded and small enveloped RNA virus of the family *Arteriviridae*, in the order *Nidovirales* (Dea, Gagnon, Mardassi, Pirzadeh, & Rogan, 2000; Meulenberg et al., 1993; Thiel et al., 1993). Genotyping studies identified two types, distinguished as the European and the North American genotype (Murtaugh, Elam, & Kakach, 1995; Nelsen, Murtaugh, & Faaberg, 1999). The disease causes reproduction disorders (such as abortions or stillbirth), respiratory disease, slow growth rates, lethargy and anorexia in all age groups (OIE, 2008; Rossow, 1998; Stadejek et al., 2002; Wensvoort, 1993; Zimmerman, Yoon, Wills, & Swenson, 1997). In Vietnam, the first outbreak was reported in the late 1990s (Nguyen, Vuong, & Vo, 2015). Since then, the PRRS virus has spread quickly across the country, contributing to serious economic losses in the pig production sector.

Previous studies suggest that the main risk factors for PRRS are the movement of infected animals between farms and the introduction of infected semen (Kittawornrat et al., 2010; Mortensen et al., 2002; Wills et al., 1997; Yaeger et al., 1993). In addition, vehicles, fomites (such as protective clothing and bedding materials) and aerosols have been associated with disease transmission (Otake, Dee, Rossow, et al., 2002; Satoshi Otake, et al., 2002; Dee, Deen, Otake, & Pijoan, 2004; Dee, Deen, Burns, Douthit, & Pijoan, 2005).

The main objective of this study was to develop various models using NAADSM to simulate the transmission of the PRRS virus between farms in Nghe An Province in Vietnam in order to inform the prevention and control of this important disease.

## 2 MATERIALS AND METHODS

### 2.1 Study location and population

**Figure 1:**
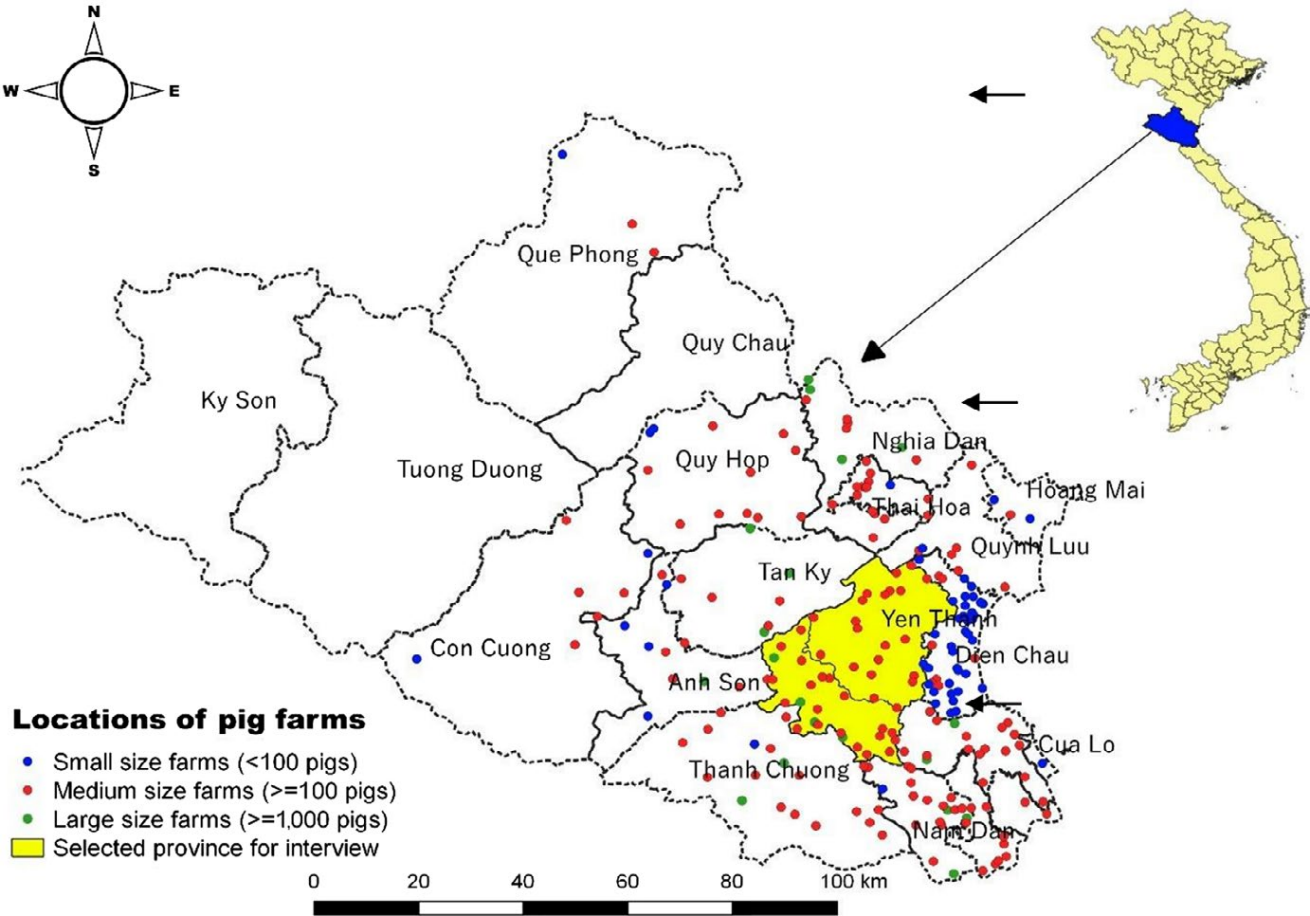
Spatial distribution of three types of pig farms at district level randomly generated by QGIS and selected province for interview [Colour figure can be viewed at http://wileyonlinelibrary.com]

Nghe An is located in the north central coast region of Vietnam and is the largest province by area in the country, with an estimated human population of 3.1 million (GSO, 2018). In order to simulate the spread of the PPRS virus between farms in NAADSM, the geographical locations and farm characteristics (such as herd size and production type) are required. Information on the number of pig farms with their herd size at the district level was obtained from local authority, though exact data on actual geographical locations of farms was not available. Random points corresponding to the number of farms per district were therefore created using QGIS (Quantum GIS development team 2012. QGIS version number 3.0.1), while longitude and latitude coordinates of these points were extracted as farm locations and then introduced into NAADSM (Figure 1).

Local data indicated a total of 232 farms in Nghe An (Table 1). Data on the number of farms were available for 18 out of 21 districts. These farms were classified into three production types: small <100 pigs; medium ≥100 and <1,000 pigs; and large farms ≥1,000 pigs (Nga, Ninh, Hung, & Lapar, 2014). Based on our criteria, the proportion of small, medium and large farms was 23.18%, 68.24% and 8.15%, respectively.

**Table 1:** Model parameters used for simulation model between pig farm spread of PRRS virus in Nghe An Province of Vietnam

Parameters	Value	References
Total farms (*n*)	232	Local authority
Small	54 (23.28%)	
Medium	159 (68.53%)	
Large	19 (8.19%)	
Transmission probability		
Direct contact (for all production types)	1	Neumann et al., (2007)
Indirect contact (for all production types)	0.1	Neumann et al., (2007)
Infectious duration		
Small	52 weeks	
Medium	52 weeks	
Large	52 weeks	
Movement control	No	
Contact distances between farms	BetaPERT (0.2,10,100)	

This study was approved by the Hanoi University of Public Health Review Board (IRB: no. 018‐186/DD‐YTCC), Vietnam. All methods were performed in accordance with the relevant guidelines and regulations.

### 2.2 Model parametrization

NAADSM requires three key parameters: (a) mean contact rates (estimated number of direct/indirect contacts between farms per week); (b) probabilities of infection transfer with each contact; and (c) contact distance between farms (Harvey et al., 2007). To obtain farm‐level data for the parametrization of the model, 60 pig farmers were interviewed in two districts (Do Luong and Yen Thanh) in Nghe An Province. The survey collected data on demographics and herd size, health status of the pig farm, number of pigs raised and contact with other farms. Farm contact information was used to estimate the “mean contact rates” for “direct contact” and “indirect contact”. The former is defined as the introduction of an infected animal from one farm to another. The latter includes the movement of people, vehicles, materials and equipment between farms.

For direct contact, data were collected on how often farmers introduced pigs on their farms and if and how often they share boars for breeding purposes. To gather information on indirect contact which potentially could transmit the PRRS virus between farms without pig movement, four questions were asked: (1) “How often did vehicles enter your farm over the last 6 months?”; (2) “How often did veterinarians/ animal health workers visit your farm over the last 6 months?; (3) How often did other farmers and traders visit your farms over the last 6 months?”; and (4) “How often did you share any equipment with other farms”. A Poisson distribution was fitted for the different model parameters, with the average number of contacts used to define λ. The distributions for mean direct and indirect contact rates were computed on a weekly basis. This period was selected as the virus can be infective for a week at 21°C (Benfield et al., 1992).

The estimated probabilities of infection transfer of the PRRS virus by direct contact was 1, while a value of 0.1 was assigned for indirect contact as these values were used in previous studies (Neumann, Morris, & Sujau, 2007; Thakur, Revie, Hurnik, Poljak, & Sanchez, 2015). No data were available to parameterize the contact distance between farms. Based on our experience, we used the BetaPERT distribution which is defined by its minimum (0.2 km), its most likely value (10 km), and its maximum (100 km) (Table 1).

We assumed that all farms were free of PRRS at the beginning of scenarios, and none of the pigs had any immunity to a new strain of virus. Furthermore, we assumed that once a single pig became infected then the whole farm was considered infectious within a week. Each farm had an equal chance to be in contact with other farms given the distance between source and recipient farms and combinations of predefined production types. Other types of indirect contact (such as airborne and fomite) were not considered. In addition, we assumed that a continuous flow (CF) system was used in all farms in our models instead of all‐in‐all‐out system (AIAO) which is not common in Vietnam.

### 2.3 Model structure and outcome

**Figure 2:**
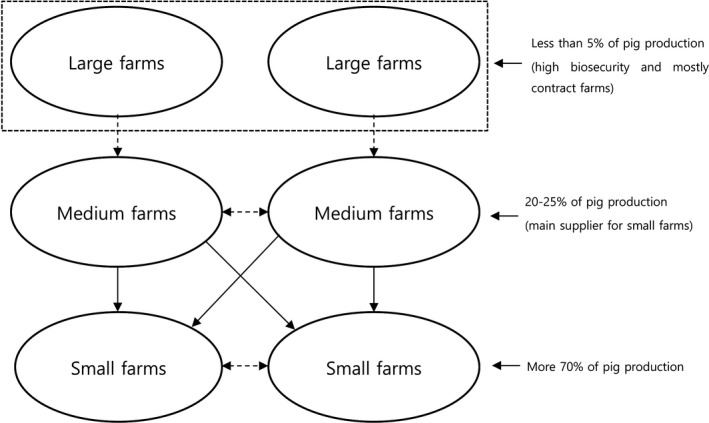
The simple characteristic of pig movement structure in Vietnam (dash arrow: rare movement)

In Vietnam, medium farms are mainly responsible for supplying piglets and weaners to small farms (Figure 2). However, there is almost zero animal movement “from small to medium”, “from small/medium farms to large farms” and between large farms. Therefore, indirect contact was only considered “from small to medium” and “from medium/large to large” farms (Table 2). We modelled 11 scenarios ‐ scenario A1 to A3 assumed a completely naïve population: scenario A1 assumed that the PRRS virus was transmitted by direct contact only, scenario A2 assumed both direct and indirect contact and scenario A3 assumed both direct and indirect contact without movement to large farms. For A1‐3 scenarios, one medium farm was randomly selected to be seeded with an infection and the same farm‐initiated infection in the subsequent iterations. The remaining farms were susceptible at the beginning of the scenarios and then became infectious until the end of the simulation. In order to evaluate the impact of an initial outbreak by production type, we generated two scenarios B1 and B2 where an initial outbreak started in a randomly selected single small (B1) or large farm (B2) via both direct and indirect contacts. Scenarios C were endemic scenarios where different proportions of medium size farms were naturally immune by 30% (scenario C1), 20% (scenario C2) and 10% (scenarios C3) following previous PRRS outbreaks. Scenarios D assumed different vaccination coverages for medium size farms: scenario D1 vaccinated by 100%; scenario D2 75%; and scenario D3 50% as those were the main pig suppliers for small farms (accounting for 70% of pig production in Vietnam). It was assumed that natural immunity and vaccine were 100% effective and conveys complete protection to the naturally immune and vaccinated herd during the studied period.

**Table 2:** Description of contact structure of pig farms used for simulation model of between farm spread of PRRS virus in Nghe An Province of Vietnam

Contact groups (Source–Destination)	Mean contact rate/week	
	Direct	Indirect
Small farms → Small farms	Poisson 0.072	Poisson 0.282
Small farms → Medium farms	‐	Poisson 0.282
Medium farms → Small farms	Poisson 0.072	Poisson 0.282
Medium farms → Medium farms	Poisson 0.073	Poisson 0.271
Medium farms → Large farms	‐	Poisson 3.5
Large farms → Medium farms	Poisson 0.073	Poisson 0.271
Large farms → Large farms	‐	Poisson 3.5

Our scenarios were simulated on a weekly basis for 52 weeks over 1,000 iterations, which was sufficient to capture the pig production life cycle (6–8 months) in Vietnam. The number of median infected pig farms and the time it took to reach peak epidemic levels were calculated for each scenario. We assumed that infected farms became infectious for long periods of time, with new susceptible animals consistently replaced during the study period. Previous studies suggest that a PRRS virus can survive in the infected farms for long periods of time (Nodelijk et al., 2000; Nodelijk, Nielen, Jong, & Verheijden, 2003). Therefore, it was assumed that these farms followed the susceptible–infectious (S‐I) transition structure, with no potential for recovery (Keeling & Rohani, 2011).

### 2.4 Sensitivity analysis

The direct contact transmission probability (DCTP) was decreased from the baseline model (contact rate:1) to 0.75, 0.5 and 0.25, respectively, while the indirect contact transmission probability (ICTP) was modified from the baseline model (contact rate: 0.1) to 0.5, 0.25 and 0.05. The number of median infected farms from each scenario was compared to the baseline model (scenario B2). In addition, we assessed the impact of the mean contact rate of indirect contact towards large farms by varying values from 3.5 to 2, 1, 0.75 and 0.5 in the model.

## 3 RESULTS

A total of 60 farmers [Female (16): male (44)] were interviewed in two districts (30 farmers/district). The mean and range of age were 51 and 25–90 years old, respectively. The survey captured data of 46 small, 11 medium and 3 large farms (See Table S1).

Of the farms enrolled in the survey, none reported that boars or equipment were shared with other farms. Table 2 summarized the baseline distributions rates (unit/week) of contact rates between the different herd size categories (small, medium and large farms), which were plugged into the model.

The modelled number of infected farms (median, 5 and 95 percentiles) by each production type is presented in Table 3. Scenarios A1 to A3 were developed with a combination of direct and indirect contacts. Scenario A2 (direct and indirect contact) showed the highest median of infected farms, affecting 90% of farms (209/232), while in scenario A1, the median number of infected farms was estimated at 21% (49/232). By including only direct contact in the models (scenario A1), the median number of infected medium size farms was dramatically decreased from 139 to 20 farms. The peak of the epidemic was reached earliest in Scenario A2, indicating rapid spread of virus among farms. Overall, the large farms must play an important role in transmitting the PRRS virus when comparing scenario A2 with other scenarios. Scenarios C were endemic scenarios where different proportion of medium size farms were naturally immunized from 30% to 10%. The naturally immune scenarios (C1 to C3) showed that the median number of infected small farms was unchanged (Table 4). However, for vaccination scenarios D where vaccine coverages were 100% (D1), 75% (D2) and 50% (D3), respectively, the median number of infected small farms was 27 (scenario D1), 41 (scenario D2) and 47 (scenario D3), indicating a reduction of infected small farms of −48%, −19.61% and −7.84%, respectively, compared to the baseline scenario (A2).

**Table 3:** Median number of infected pig farms and time required to reach the peak epidemic under assumptions of various direct and indirect contacts

Scenario	Contact information	No. of infected farms: median (5 and 95 percentiles)				Week to peak epidemic
		Overall	Small	Medium	Large	
A1	Direct contact	49 (14–100)	30 (10–44)	20 (1–59)	0	45
A2	Direct and indirect contact	209 (191–219)	51 (48–53)	139 (122–148)	19 (19–19)	32
A3	Direct and indirect contact (no contact to large farms)	99 (156–30)	42 (21–50)	56 (7–107)	0	38

**Table 4:** Median number of infected pig farms after the medium size farms were immunized under different scenarios

Scenario	Contact information	No. of infected farms: median (5 and 95 percentiles)				% change in median outcome of small farms compared to baseline
		Overall	Small	Medium	Large	
A2	Direct and indirect contact	209 (191–219)	51 (48–53)	139 (122–148)	19 (19–19)	N/A
C1	30% naturally immune	160 (142–172)	51 (46–53)	91 (75–101)	19 (19–19)	0%
C2	20% naturally immune	173 (157–184)	51 (47–53)	104 (89–113)	19 (19–19)	0%
C3	10% naturally immune	195 (177–205)	52 (48–54)	125 (109–134)	19 (19–19)	0%
D1	100% vaccination	46 (25–61)	27 (6–42)	0	19 (19–19)	−47.71%
D2	75% vaccination	77 (61–89)	41 (28–47)	17 (10–25)	19 (19–19)	−19.61%
D3	50% vaccination	115 (96–129)	47 (40–51)	50 (36–61)	19 (19–19)	−7.84%

The number of median infected farms for scenario B1 was much lower than scenarios A2 and B2, where a single outbreak occurred in a small farm (See Figure S1). For each scenario, the median number of infected small farms was 14 (scenario B1), 51 (scenario A2) and 51 (scenario B2). In addition, the median number of infected medium farms was 0 (scenario B1), 139 (A2) and 138 (B3), respectively.

The sensitivity analysis showed that the number of median infected farms was very sensitive to the DCTP (Table 5), suggesting that changes in this rate had a huge impact on our results in the models. For example, a reduction of DCTP by −75% (from 1 to 0.25) had resulted in a decrease of the number of median infected farms by −44.50%. However, changes in ICTP had less impact on our outcomes of the models. In particular, an increase of ICTP by 400% (from 0.1 to 0.5) had resulted in an increase of the number of median infected farms by only 10.53% (from 209 to 231). Additional sensitivity analysis was conducted for indirect contact from medium/large to large farms. It showed that a reduction in mean indirect contact by −78.57% (from 3.5 to 0.75) had little impact on our outcomes, resulting in a decrease of median number of infected farms by −12.44% (See Table S2). However, mean indirect contact of 0.5 showed a large reduction in the number of median infected farms (−48.80%; 209–>107). Of note, was a sharp decrease in the median number of infected large farms from 19 to 3.

**Table 5:** Sensitivity analysis of the median epidemic size of simulated PRRS outbreaks to direct and indirect contact transmission probability in a population of 232 pig farms

Scenarios	Parameters		± % change of parameters		Epidemic size median (5 and 95 percentile)	% change in median outcome compared to baseline
	DC Transmission probability	IC Transmission probability	DC Transmission probability	IC Transmission probability		
Baseline	1	0.1	N/A	N/A	209 (191–219)	N/A
DC change 1	0.75	0.1	−25%	N/A	192 (167–208)	−8.13%
DC change 2	0.5	0.1	−50%	N/A	159 (126–182)	−23.92%
DC change 3	0.25	0.1	−75%	N/A	116 (87–142)	−44.50%
Baseline	1	0.1	N/A	N/A	209 (191–219)	N/A
IC change 1	1	0.5	N/A	400%	231 (228–231)	110.53%
IC change 2	1	0.25	N/A	150%	227 (223–230)	108.61%
IC change 3	1	0.05	N/A	−50%	183 (138–206)	−12.92%

Abbreviations: DC, direct contact; IC, indirect contact.

## 4 DISCUSSION

To our understanding, this was the first study to evaluate the spread of PRRS virus between farms via direct and indirect contacts in Vietnam. Using real data from the household survey, credible parameters for mean contact rates between different farms were estimated. Furthermore, the number of pig farms by production types used in our models was based on local data, adding to the robustness of the model.

Scenario A2 included both direct and indirect contact while scenario A1 only had direct contact. As a result, scenario A2 showed that the number of median infected farms was the highest and quickest to reach the peak epidemic. In addition, scenarios A2 and B2 had relatively higher number of median infected farms. Our findings suggest that medium and large farms may play an important role in the transmission of PRRS virus in Nghe An. In general, large farms are isolated and mostly managed by big commercial companies in Vietnam. For this reason, certain direct contact routes were not taken into account as in practice, and there is no animal movement towards the large farms. There is a hierarchical structure of animal movement from medium to small farms. Therefore, no animal movement from small to medium farms was considered as the small farms are the last stage of pig's life cycle in Vietnam, which are brought to the slaughterhouses. Our survey showed that the indirect contact rate in large farms was considerably higher than small and medium farms, likely reflecting the fact that there are frequent vehicle and human movements for pig sales and farm managements in comparison with small and medium scale farms.

Our models showed that the transmission route to large farms via indirect contact could have a significant impact on our results. Given the strict restrictions in place, however, it is unlikely that the large farms allow the sharing of vehicles and human movement with other farms. Therefore, the medium farms need to be targeted to efficiently reduce/prevent the transmission of the PRRS virus to small‐scale farms, which account for 70% of the total production in Vietnam (Lapar, Binh, & Ehui, 2003). Immunization scenarios showed that a high proportion of medium size farms should be vaccinated in order to reduce the transmission to small farms under the Vietnamese pig production system. In general, medium size farmers are relatively wealthier and more likely to invest in vaccines and biosecurity rather than small farmers. In order to promote update of vaccinations, however, incentives (such as a vaccine subsidy) for medium size farmers may be needed. It could be the most cost‐effective control and prevention strategy for pig diseases in Vietnam.

The model assumed that the whole farm became infectious when one pig was infected from the farm, which was reasonable as it is unlikely that the PRRS virus would die out without onward transmission of diseases to other pigs in the farms. Similarly, many studies have suggested that R_0_ of PRRS virus was higher than 1 (Charpin et al., 2012; Nodelijk et al., 2000; Zhang, Kono, & Kubota, 2014). Some studies have suggested that PRRS virus has a high degree of genetic and antigenic variability (Chang et al., 2002; Tian et al., 2007), which may result in different levels of cross‐protection of different morbidities. However, we assumed that there was no emergence of a new strain or multiple strains circulating during the modelled study period. Simulation models are approximate imitations of real‐world, so it is necessary to validate how our models represent the dynamic of the disease in a population. However, we were not able to validate our models due to lack of real outbreak data in Nghe An.

The main limitation of our study was that our direct contact transmission probability was considered to be 1 which may have led to an over‐estimation as some of farms may use AIAO production system, so virus transmission rate might be less than 1. The importance of the transmission probability was also confirmed in the sensitivity analysis. Values used were based on studies by others (Neumann et al., 2007; Thakur et al., 2015), but may need to be adjusted for Vietnam as more evidence becomes available.

This study is the first attempt to quantify the indirect contact rates from the survey. Our estimates are mainly based on vehicle and human movements and may have underestimated the number of infected farms in practice. Some studies have suggested that the virus could be disseminated by aerosol and fomite transmissions (Pirtle & Beran, 1996; Otake, Dee, Rossow, et al., 2002; Dee, Otake, Oliveira, & Deen, 2009; Le, Poljak, Deardon, & Dewey, 2012). Another study showed that some avian species (such as chickens and ducks) are susceptible to PRRS virus via natural exposure and may play a role in the transmission of disease to pigs (Zimmerman et al., 1997). It may be possible that the virus can be transmitted from poultry to pigs as mixed livestock farming systems are very common in small farms in Vietnam. These potential risk factors could therefore be added as the indirect contact. Lastly, we did not take into account of the prevention and control strategies in the current models, which were likely to have overestimated the disease transmission between farms. One study suggested that PRRS transmission was significantly associated with location of the farms, farm management, human and animal contact (Truong & Gummow, 2014).

A few simulation studies on the spread of PRRS virus have been conducted in New Zealand and Canada using similar software (Neumann et al., 2007; Thakur et al., 2015). The mean direct contact rates used in New Zealand (0.064–0.21/week) and Canada (0.01–0.51/week) were slightly higher than our study (0.072–0.073/week). However, indirect contact rate in our study was considerably higher (0.271–3.5/week) than other studies conducted in New Zealand (0.15–0.5/week) and Canada (0.0007–0.16/week). This is mainly due to the fact that our indirect contact took into account, both vehicle and human movements. In addition, it could be possible that there is a relatively low level of awareness in biosecurity, which would allow movement without restriction. Having compared practices against those in other countries, our findings suggest that it is necessary to reduce a number of indirect contacts between farms.

It is essential to educate farmers to have an isolation area for newly pig arrivals and an AIAO system in order to prevent the spread of disease on farms. Based on our observations, these are not properly implemented in small farms due to the low level of awareness in biosecurity and for economic reasons. In addition, it is important to control the movement of infected or high‐risk farms as this would help stop the spread of PRRS virus. The PRRS virus vaccine has been introduced to farmers in Vietnam, but is mainly used in large‐scale pig farms. They are not in use, in small‐scale farms due to economic constraints and the absence of incentives to promote the use of PRRS vaccination, since pigs can be traded without restrictions.

Despite the discussed limitations and difficulties to validate our model, it provides valuable insight into how PRRS virus can be spread between farms via direct and indirect contact. Our model could aid decision makers in directing resources towards the prevention and reduction of transmission of PRRS virus in farms. The model can be easily adjusted to other provinces or countries in the region with modified parameters.

## CONFLICT OF INTEREST

The authors declare that they have no competing interests.

## AUTHOR CONTRIBUTIONS

H.S.L, K.K.T, and B.W. designed research: H.S.L, V.B.N, N.A. and D.M. performed research; H.S.L and K.K.T. analysed data; H.S.L, K.K.T. and B.W. wrote the paper.
