# Supplementary material for: Simulation of control scenarios of porcine reproductive and respiratory syndrome in Nghe An Province in Vietnam
Source: Transbound Emerg Dis. 2019 Jul 7;66(6):2279–87. doi: 10.1111/tbed.13278 (PMC6899877; doi:10.1111/tbed.13278)
Supplement: Supplementary file 1 [file TBED-66-2279-s001.docx]

Supplementary 1. Summary of survey data for pig farms in Nghe An province of Vietnam

| Parameters | Value |
| --- | --- |
| Total farms (n) | 60 |
| Small | 46 (76.67%) |
| Medium | 11 (18.33%) |
| Large | 3 (5.0%) |
| Gender (n) |  |
| Female | 16 (26.67%) |
| Male | 44 (73.33%) |
| Age (year) |  |
| <30 | 2 (3.33%) |
| 30-39 | 4 (6.67%) |
| 40-49 | 23 (38.33%) |
| 50-59 | 21 (35.0%) |
| ≥60 | 10 (16.67%) |
|  |  |

Supplementary 2. Distribution of the number of infected farms where an initial outbreak started in a randomly selected single small (B1), medium (A2) and large farm (B2) via both direct and indirect contacts

Number of infected farms

Supplementary 3. Sensitivity analysis of the median epidemic size of simulated PRRS outbreaks to mean contact rate of indirect contact from medium/large to large pig farms

| Scenarios | Parameters | | |  | ± % change of parameters | | | Epidemic Size Median(5 and 95 percentile) | % change in median outcome compared to baseline |
| --- | --- | --- | --- | --- | --- | --- | --- | --- | --- |
|  | DC^a^ Transmission probability | IC^b^  Transmission probability | Mean contact rate from M/L to L |  | DC  Transmission probability | IC  Transmission probability | Mean contact rate from M^c^/L^d^ to L |  |  |
| Baseline | 1 | 0.1 | 3.5 |  | N/A | N/A | N/A | 209 (191-219) | N/A |
| MCR 1 | 1 | 0.1 | 2 |  | N/A | N/A | -42.86% | 204 (171-218) | - 2.39% |
| MCR 2 | 1 | 0.1 | 1 |  | N/A | N/A | -71.43% | 191 (129-213) | - 8.61% |
| MCR 4 | 1 | 0.1 | 0.75 |  | N/A | N/A | -78.57% | 183 (103-209) | - 12.44% |
| MCR 3 | 1 | 0.1 | 0.5 |  | N/A | N/A | -85.71% | 107 (37-172) | - 48.80% |

^a^DC: direct contact; ^b^IC: indirect contact; N/A: not applicable; ^c^M: Medium; ^d^L: Large
